# Joint inversion of transient electromagnetic and radiomagnetotelluric data for enhanced subsurface characterization

**DOI:** 10.1038/s41598-025-10959-2

**Published:** 2025-07-15

**Authors:** Ismael M. Ibraheem, Pritam Yogeshwar, Fereydoun Sharifi, Rainer Bergers, Bülent Tezkan

**Affiliations:** 1 Institute of Geophysics and Meteorology, University of Cologne, Pohligstrasse 3, 50969 Cologne, Germany; 2 Leibnitz Institute for Applied Geophysics (LIAG), Stilleweg 2, 30655 Hanover, Germany

**Keywords:** Radiomagnetotelluric (RMT), Transient electromagnetics (TEM), Joint inversion, Landfill characterization, Subsurface geophysical imaging, Geophysics, Environmental impact

## Abstract

Joint inversion of geophysical data offers a robust means to improve the resolution and reliability of subsurface models, particularly when integrating methods with complementary depth sensitivities. This study presents a 1D joint inversion methodology for combining Transient Electromagnetic (TEM) and Radiomagnetotelluric (RMT) data, two complementary electromagnetic methods that provide insights into different depth ranges of the subsurface. The approach is validated through synthetic modeling and applied to a landfill site to demonstrate its practical utility. Results confirm that joint inversion significantly reduces model ambiguity and enhances the delineation of key structures, including the extent of the waste body. These findings show the methodological advantages of the proposed joint inversion strategy in resolving complex, heterogeneous environments, and highlight its potential for broader applicability in environmental and engineering geophysics.

## Introduction

Geophysical methods such as gravity, magnetic, electrical, electromagnetic, and seismic surveys each provide unique insights into the Earth’s physical properties. However, they often face challenges related to non-uniqueness and limited resolution. To overcome these limitations and improve subsurface interpretation, joint inversion integrates multiple geophysical datasets within a unified mathematical framework, leveraging their complementary nature to construct more accurate and reliable subsurface models^1,2^. This approach enhances the consistency of earth models, providing a more comprehensive understanding of subsurface structures and improving the precision of geophysical interpretations.

The concept of joint inversion in geophysics was first introduced in the 1970s through the work of Vozoff and Jupp^3^ who integrated magnetotelluric (MT) and vertical electrical sounding (VES) data. Since then, the technique has been widely applied to various geophysical datasets, including seismic, gravity, magnetic, electric, and electromagnetic methods^4–12^. Colombo and De Stefano^13^ advanced this approach by demonstrating the simultaneous joint inversion of seismic, gravity, and electromagnetic data, highlighting its ability to reduce uncertainties and provide a more comprehensive understanding of reservoir properties. Also, Candansayar and Tezkan^14^ developed a 2D joint inversion algorithm for radiomagnetotelluric and direct current resistivity data, which was successfully applied to identify an active fault near Kerpen, Germany. Further advancements in joint inversion methodologies have expanded its applications. Lin and Zhdanov^15^ introduced a joint inversion method of gravity and magnetic data using multinary transformation and Gramian constraints to accurately identify shapes, locations, and properties of anomalous targets.

When geophysical methods involve fundamentally different physical parameters, such as seismic velocity and electrical resistivity, structural or statistical coupling strategies become essential. One such approach is the cross-gradient constraint^16^ which promotes alignment of structural features between datasets without assuming direct parameter correlation. This strategy was originally introduced for the joint inversion of electrical resistivity and seismic velocity data^16^. It has been widely used in 2D and 3D studies, including the integration of marine controlled-source electromagnetic (CSEM) and seismic refraction data in offshore Borneo, yielding improved imaging of geohazards and overburden anomalies^12^. More recently, Penta de Peppo et al.^17^ developed a cross-gradient joint inversion algorithm for electrical and seismic tomography on structured meshes with non-flat topography. Their method was validated using a novel cross-gradient index and fuzzy c-means clustering analysis. Another strategy is Variation of Information (VoI) joint inversion, which minimizes structural dissimilarity through statistical metrics rather than enforcing similarity directly. This technique has been applied to magnetotelluric–gravity^7^ and gravity–magnetic^18^ joint inversions, showing enhanced structural detail and improved model coherence.

More recently, Mongabadi et al.^19^ applied a cross-gradient constrained joint inversion of magnetometry and DC resistivity data to enhance the reconstruction of geological features. Kästle et al.^2^ developed an innovative joint inversion approach combining surface-wave and teleseismic body-wave travel times, significantly improving crustal and mantle imaging in the Alpine region. Similarly, Lee et al.^20^ jointly inverted Rayleigh wave phase velocity and its horizontal-to-vertical amplitude ratio into a 2-D shear wave velocity to image the deformation belt of western Hispaniola Island in Haiti. Ai et al.^21^ introduced the hunger games search (HGS) algorithm for the joint inversion of multimode Rayleigh wave dispersion data to estimate glacier ice thickness and subglacial properties. Fang et al.^22^ further advanced joint inversion techniques by introducing an improved 3D joint inversion of gravity and magnetic data using deep learning, achieving higher accuracy in subsurface density and susceptibility modeling. These studies show the continuous evolution of joint inversion, demonstrating its growing significance in geophysical research and subsurface characterization.

However, such coupling strategies are primarily relevant for joint inversion scenarios involving disparate physical properties. In contrast, the TEM and RMT methods both measure the Earth’s subsurface resistivity and thus share a direct physical parameter. This inherent compatibility eliminates the need for cross-gradient constraints or petrophysical coupling, allowing a direct joint inversion framework where both datasets are integrated through their common resistivity sensitivity. Individually, TEM and RMT each offer distinct strengths and limitations. TEM is effective and well-suited for deep imaging but lacks resolution in shallow layers, whereas RMT provides high-resolution data for shallow structures but has limited depth of investigation. Due to their complementary depth sensitivities, integrating TEM and RMT data can enhance subsurface imaging. Tezkan et al.^23^ successfully delineated the lateral and vertical boundaries of a waste deposit in Ossendorf, Cologne, through a combined interpretation of TEM and RMT data. However, no studies have specifically applied joint inversion of these methods for landfill characterization. Widodo^24^ utilized these two methods to image shallow and deep fault structures in the Volvi Basin, northern Greece, while Widodo et al.^25^ used RMT and TEM surveys across eight profiles in the Mygdonian Basin, northeast of Thessaloniki, to map subsurface conductivity variations and resolve a complex 3D fault system. Sequential and joint inversions revealed a normal fault structure with depths up to 200 m, aligning well with borehole data and improving geological interpretations, particularly in identifying graben features and refining local fault geometry.

Several studies have developed joint inversion approaches for various combinations of EM methods, including TEM and MT^5,26,27,^ RMT and TEM^28,29^, and integrated airborne and ground-based EM data^30^. While these studies laid important groundwork, many employed separate inversion routines or relied on heuristic coupling strategies. Notably, despite the widespread individual use of RMT and TEM for near-surface studies, a comprehensive 1D joint inversion framework for RMT and TEM has not been previously published. In this study, we present a novel implementation of a flexible forward and joint inversion framework using the EMUPLUS code developed at the University of Cologne that supports both Occam and Levenberg–Marquardt algorithms, tailored specifically for the RMT–TEM combination. We further introduce a formal resolution and importance analysis that evaluates the relative sensitivity contributions of each dataset, thereby enhancing model interpretability. Synthetic tests explore the complementarity of the two methods, while a field application to a contaminated landfill demonstrates the practical utility of the joint inversion approach. These advancements establish a novel, reproducible methodology for high-resolution near-surface characterization using complementary EM datasets.

## Materials and methods

In the joint inversion of TEM and RMT data, both methods are sensitive to a common subsurface parameter (i.e., electrical resistivity). This shared sensitivity enables the use of straightforward joint inversion frameworks, where a single resistivity model is updated to fit both datasets simultaneously. In contrast, joint inversion of geophysical methods that involve different physical parameters, such as seismic (velocity) and gravity (density), requires more complex strategies. These include structural coupling or petrophysical relationships to link the models, since each method responds to fundamentally different properties of the subsurface.

### TEM and RMT techniques

**Fig. 1:**
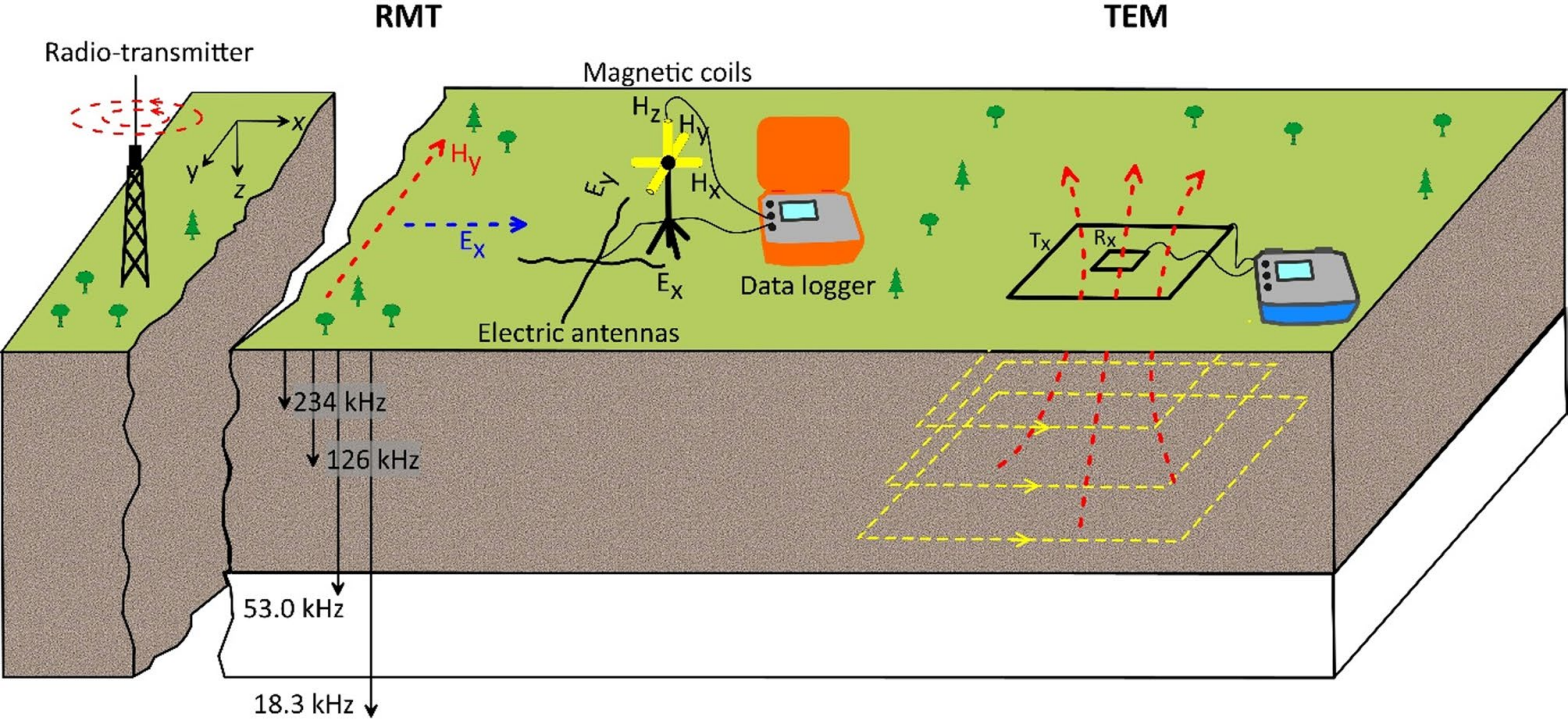
A simplified schematic illustrating the field setup for TEM and RMT techniques. For the RMT technique, the diagram schematically shows frequency-dependent skin depth. Black squares indicate the transmitter (Tx) and receiver (Rx) loops used in the TEM method, while the dashed yellow lines beneath them represent the evolution of induced eddy currents at increasing times after current turnoff. The red dashed lines refer to magnetic fields.

TEM and RMT are two widely used electromagnetic methods in geophysical exploration. TEM is an active time-domain method that employs a transmitter loop (Tx) on the surface to create a primary electromagnetic field. When the current is abruptly cut off, it induces eddy currents in the subsurface, generating a secondary magnetic field. This field is recorded by a surface receiver loop (Rx), and the decay of the induced voltage over time is analysed to reveal the subsurface’s electrical resistivity^31^. TEM’s strength lies in its ability to penetrate deep layers, making it useful for mapping subsurface structures at deep levels^32–34^. It is often used in groundwater exploration^35–37^ and environmental studies^38–42^. However, the resolution of the method is relatively low for shallow layers, leading to challenges in accurately characterizing near-surface anomalies^23^. A schematic diagram illustrating the field setup and arrangement of equipment for TEM and RMT techniques is depicted in Fig. 1.

The RMT method is a passive, frequency-domain electromagnetic technique that utilizes natural and artificial electromagnetic fields from distant radio transmitters, operating in the frequency range of 10 kHz to 1 MHz^43^. Known for its efficiency and simplicity in field operations, RMT measures the electric field (E-field) using two electrical dipoles and the magnetic field (H-field) using three induction coils in perpendicular directions (Fig. 1). These measurements capture the amplitude and phase of electromagnetic signals as they interact with the subsurface. The data is then processed to derive apparent resistivity and phase, enabling the creation of high-resolution resistivity models for shallow structures. RMT produces high-resolution images of shallow subsurface structures, making it particularly effective for identifying different subsurface targets such as contaminated areas^44,45,^ landfill sites^23,^ groundwater resources^46,^ landslides^47,48^, and fault or fracture zones^49,50^. However, while RMT provides high resolution for near-surface layers, it is limited in its ability to image deeper structures due to its relatively shallow depth of investigation. This limitation is particularly noticeable in conductive subsurface environments, such as waste sites, where deeper boundaries may not be imaged. Furthermore, RMT is not suitable for use in remote areas with insufficient transmitters^50^.

### Forward modelling

#### TEM forward modelling

For 1D forward modelling of central loop TEM data, the vertical component of the magnetic field $\:{B}_{z}\:$is calculated using the following integral^51^:

1 $$\:{B}_{z}=\frac{{\mu\:}_{0}\widehat{m}}{2}{\int\:}_{0}^{\infty\:}\left({e}^{-{\widehat{\alpha\:}}_{0}\left(z+h\right)}+{R}_{TE}.{e}^{{\widehat{\alpha\:}}_{0}\left(z-h\right)}\right)\frac{{\lambda\:}^{2}}{{\widehat{\alpha\:}}_{0}}{J}_{1}\left(\lambda\:r\right)d\lambda\:$$

where $\:\widehat{m}$ denotes the magnetic dipole moment of the transmitter loop, ${\mu\:}_{0}$ is the permeability of free space $(4{\uppi\:}\:\times\:\:10^-7\:\text{H}/\text{m})$, *r* is the loop radius, $\:{\widehat{\alpha\:}}_{0}$ is the characteristic admittance of free space, z and h are the transmitter and receiver loops altitude, $\:{R}_{TE}$ is a reflection coefficient at the surface of the L-layered half-space model, $\:\lambda\:$ is a wavenumber, and $\:{J}_{1}$ is a Bessel function of order 1 of the first kind.

The reflection coefficient $\:{R}_{TE}\:$is given by,

2 $$\:{R}_{TE}=\:\frac{\lambda\:-\:{\beta\:}_{1}}{\lambda\:+\:{\beta\:}_{1}}$$

The recursion $\:{\beta\:}_{l}$ for the *l*-th layer is:

3 $$\:{\beta\:}_{l}=\:{\widehat{\alpha\:}}_{l}\frac{{\beta\:}_{l+1}+{\widehat{\alpha\:}}_{l}\text{tanh}\left({\widehat{\alpha\:}}_{l}.{d}_{l}\right)}{{\widehat{\alpha\:}}_{l}+{\beta\:}_{l+1}\text{tanh}\left({\widehat{\alpha\:}}_{l}.{d}_{l}\right)}$$

with

4 $$\:{\widehat{\alpha\:}}_{l}=\:\sqrt{{\lambda\:}^{2}+\frac{i\omega\:\mu\:}{{\rho\:}_{l}}}$$

where $\:\omega\:$, $\:{\rho\:}_{l}$, and $\:{d}_{l}$ stand for angular frequency, resistivity and thickness of *l*-th layer, respectively. For a half-space, a value of $\:{\beta\:}_{L}=\:{\widehat{\alpha\:}}_{L}\:$is chosen for the last layer which provides a boundary condition to initiate the recursion.

To solve the Eq. (1) in frequency domain, the Fast Hankel Transform method is used. Subsequently, the inverse Fourier transform is employed to calculate the time derivative of a magnetic flux for a step current^51^.

5 $$\:{B}_{z}\left(t\right)=\frac{2}{\pi\:}{\int\:}_{0}^{\infty\:}Re\left(\frac{{B}_{z}\left(\omega\:\right)}{\omega\:}\right)\text{sin}\left(\omega\:t\right)d\omega\:\:$$

6 $$\:\frac{{\partial\:B}_{z}\left(t\right)}{\partial\:t}=\sqrt{\frac{2}{\pi\:t}}{\int\:}_{0}^{\infty\:}Re\left({B}_{z}\left(\omega\:\right)\right)\sqrt{\omega\:}{J}_{-\frac{1}{2}}\left(\omega\:t\right)d\omega\:\:$$

7 $$\text{where}\, \:{J}_{-\frac{1}{2}}\left(\omega\:t\right)\:=\:\sqrt{\frac{2}{\pi\:\omega\:t}}\text{cos}\left(\omega\:t\right)$$

The late time apparent resistivity is then derived using^52^,

8 $$\:{\rho\:}_{a}^{l}=\:{\left(\frac{{I}^{2}{r}^{4}}{{20}^{2}\pi\:{t}^{5}}\right)}^{1/3}{\mu\:}_{0}{\left(-\frac{\partial\:{H}_{z}}{\partial\:t}\right)}^{-2/3}$$

where *I* is transmitter current (A), *t* is time (s), and $\:\frac{\partial\:{H}_{z}}{\partial\:t}$ stands for time derivative of induced magnetic field (A/m²).

#### RMT forward modelling

For 1-D modelling over a layered half-space model in the far-field zone, the commonly used apparent resistivity $\:{\rho\:}_{a}\:$and phase $\:{\phi\:}_{a}\:$are calculated as follows^43^,

9 $$\:{\rho\:}_{a}=\frac{1}{\omega\:{\mu\:}_{0}}{\left|Z\right|}^{2}\:$$

10 $$\:{ \varphi }_{a}={tan}^{-1}\left(\frac{Im\left({E}_{X}/{H}_{y}\right)}{Re\left({E}_{X}/{H}_{y}\right)}\right)\:$$

where Z stands for complex impedance at the surface given by,

11 $$\:{Z}_{l}=i\omega\:{C}_{l}\:$$

where $\:{C}_{l}$ is the Schmucker-Weidelt complex transfer function for *l*-th layer^53,54^ computed using Wait’s recursion formula^55^:

12 $$\:{C}_{l}=\:\frac{{{k}_{l}C}_{l+1}+{k}_{l}\text{tanh}\left({k}_{l}{d}_{l}\right)}{{k}_{l}(1+{{k}_{l}C}_{l+1}\text{tanh}\left({k}_{l}{d}_{l}\right))}$$

where $\:{k}_{l}$ is a complex wavenumber of each layer and calculated using:

13 $$\:{k}_{l}=\:\sqrt{\frac{i\omega\:{\mu\:}_{0}}{{\rho\:}_{l}}}$$

and the complex transfer function of last layer, L, is given by:

14 $$\:{C}_{L}=\:\frac{1}{{k}_{L}}$$

### Inverse modelling

For the inversion of TEM and RMT data, we use different schemes, including Occam, Levenberg-Marquardt (LM) method as well as a joint inversion using both of these algorithms, implemented in the EMUPLUS code. In this procedure, Occam’s inversion provides a starting model for LM inversion. Furthermore, by comparing the results of Occam’s inversion using roughness R1 and R2, we gain insight into the depth of investigation and coverage of acquired data in each TEM/RMT station.

In the inversion schemes, the objective function of the form^56^,

15 $$\Phi = \Phi _{d} + ~\alpha \Phi _{m} = \left\| {\user2{W}_{\user2{d}} \left( {\user2{d} - F\left( \user2{m} \right)} \right)} \right\|_{2}^{2} + ~\alpha \left\| {\user2{W}_{m} \left( {\user2{m} - \user2{m}_{\user2{r}} } \right)} \right\|_{2}^{2}$$

is minimized with regard to the model parameter vector ***m***. where, ***d*** stands for the data vector, *F(****m****)* is the forward response, $\:{\varvec{W}}_{\varvec{d}}$ and $\:{\varvec{W}}_{m}$ are data and model weighting matrices, $\:\alpha\:$ is a regularization parameter, and $\:{\varvec{m}}_{\varvec{r}}$ is a reference model obtained from prior information. The data weighting matrix is a diagonal matrix $\:{\varvec{W}}_{\varvec{d}\varvec{i}\varvec{i}}=\frac{1}{{\sigma\:}_{i}}$, where $\:{\sigma\:}_{i}$ is the standard deviation of the observation error associated with the i-th data point. The quality of the data fit is assessed using the Chi-value ($\:\chi\:)$,

16 $$\:\chi\:=\sqrt{\frac{{\Phi\:}_{d}}{N}}=\:\sqrt{\frac{1}{N}\sum\:_{i=1}^{N}\frac{{\left({d}_{i}-{F}_{i}\left(\varvec{m}\right)\right)}^{2}}{\delta\:{d}_{i}^{2}}}$$

where, $\:{d}_{i}\:$ refers to the observed data, $\:{F}_{i}\left(\varvec{m}\right)$​ represents the calculated data, $\:\delta\:{d}_{i}$ stands for data-error, and N is the total number of data points. A χ value of 1 corresponds to an ideal data fit, reflecting the error margin. Values of χ less than 1 suggest overfitting, while values greater than 1 point to insufficient data fit^57^.

#### Occam’s inversion

The 1D Occam’s inversion of TEM and RMT data is based on a fixed number of layers (≈ 40) with a predefined thicknesses and then iterates to derive the resistivity of each layer to fit the observed data. To achieve a balance between model flexibility and smoothness, the roughness constraints R_1_ and R_2_ are applied^58^,

17 $$\:{R}_{1}=\int\:{\left(\frac{d\rho\:}{dz}\right)}^{2}dz\\{R}_{2}=\int\:{\left(\frac{{d}^{2}\rho\:}{{d}^{2}z}\right)}^{2}dz$$

which in finite difference discrete form are given as,

18 $$\begin{gathered} \:R_{1} = \left[ {\begin{array}{*{20}c} 0 & {\: \cdots \:} & {\: \cdots \:} & {\: \cdots \:} & {\: \cdots \:} & {\:0} \\ {\: - 1} & {\:1} & {\:0} & {\: \cdots \:} & {\: \cdots \:} & {\: \vdots } \\ {\:0} & {\: - 1} & {\:1} & {\:0} & {\: \cdots \:} & {\: \vdots } \\ {\: \vdots } & {\: \cdots \:} & {\: \ddots \:} & {\: \ddots \:} & {\: \cdots \:} & {\: \vdots } \\ {\: \vdots } & {\: \cdots \:} & {\: \cdots \:} & {\: \ddots \:} & {\: \ddots \:} & {\:0} \\ {\:0} & {\: \cdots \:} & {\: \cdots \:} & {\:0} & {\: - 1} & {\:1} \\ \end{array} } \right] \hfill \\ \:R_{2} = \left[ {\begin{array}{*{20}c} 0 & {\: \cdots \:} & {\: \cdots \:} & {\: \cdots \:} & {\: \cdots \:} & {\:0} \\ {\: - 1} & {\:1} & {\:0} & {\: \cdots \:} & {\: \cdots \:} & {\: \vdots } \\ {\: - 1} & {\:2} & {\: - 1} & {\:0} & {\: \cdots \:} & {\: \vdots } \\ {\:0} & {\: - 1} & {\:2} & {\: - 1} & {\:0} & {\: \vdots } \\ {\: \vdots } & {\: \cdots \:} & {\: \ddots \:} & {\: \ddots \:} & {\: \ddots \:} & {\:0} \\ {\:0} & {\: \cdots \:} & {\:0} & {\: - 1} & {\:2} & {\: - 1} \\ \end{array} } \right] \hfill \\ \end{gathered}$$

Using the roughness matrix as a model constraint, the model update is calculated as follows^59^:

19 $$\:{m}_{k+1}\left(\alpha\:\right)={\left(\alpha\:{R}_{i}^{T}+{\left({\varvec{w}}_{d}\varvec{J}\right)}^{T}{\varvec{w}}_{d}\varvec{J}\right)}^{-1}{\left({\varvec{w}}_{d}\varvec{J}\right)}^{T}{\varvec{w}}_{d}(\varvec{d}-F\left({\varvec{m}}_{\varvec{k}}\right)\:+\:\varvec{J}{\varvec{m}}_{k})$$

where R_i​_ denotes the roughness regularization operator where i equals 1 or 2, and J is the Jacobian matrix expressed as,

20 $$\:\:J_{{ij}} = \left[ {\frac{{\partial \:F_{i} }}{{\partial \:m_{j} }}} \right] = \:\left[ {\begin{array}{*{20}c} {\frac{{\partial \:F_{1} }}{{\partial \:m_{1} }}} & {\:\frac{{\partial \:F_{1} }}{{\partial \:m_{2} }}} & {\: \cdots \:} & {\:\frac{{\partial \:F_{1} }}{{\partial \:m_{M} }}} \\ {\:\frac{{\partial \:F_{2} }}{{\partial \:m_{1} }}} & {\:\frac{{\partial \:F_{2} }}{{\partial \:m_{2} }}} & {\: \cdots \:} & {\:\frac{{\partial \:F_{2} }}{{\partial \:m_{M} }}} \\ {\: \vdots } & {\: \vdots } & {\: \cdots \:} & {\: \vdots } \\ {\:\frac{{\partial \:F_{N} }}{{\partial \:m_{1} }}} & {\:\frac{{\partial \:F_{N} }}{{\partial \:m_{2} }}} & {\: \cdots \:} & {\:\frac{{\partial \:F_{N} }}{{\partial \:m_{M} }}} \\ \end{array} } \right]$$

where, *M* and *N* stand for the dimension of model and data vectors, respectively.

The regularization parameter $\:\alpha\:$ in each iteration is determined according to the discrepancy principal, ensuring that the smoothest model with the best data misfit ($\:{\varPhi\:}_{d}$) is obtained using a line search strategy^60^.

#### Levenberg–Marquardt inversion

In the context of LM algorithm, we constrain the number of layers based on the turning points of recovered resistivity profile using Occam’s inversion and use the corresponding resistivity and thickness as starting model for LM inversion. The model update in LM scheme is calculated as,

21 $$\:{\varvec{m}}_{k+1}={\varvec{m}}_{k}+\:\gamma\:\delta\:\varvec{m}$$

where $\:\delta\:\varvec{m}$ is a search direction given as,

22 $$\:\delta\:\varvec{m}={\left({\varvec{J}}^{T}{\varvec{w}}_{\varvec{d}}^{2}\varvec{J}+\alpha\:\:\varvec{I}\right)}^{-1}{\varvec{J}}^{T}{\varvec{w}}_{\varvec{d}}^{2}(\varvec{d}-F\left({\varvec{m}}_{\varvec{k}}\right))$$

and $\:\gamma\:$ is the step length of the move toward the next iteration that is obtained using line search strategy. I is the identity matrix and α balances stability and convergence^61,62^.

The Marquardt inversion employs a damping factor as a numerical stabilizer, which is iteratively optimized to minimize misfit while maintaining computational stability through the matrix term $\:\alpha\:\varvec{I}$ in the normal equation. This approach prioritizes rapid convergence without explicit smoothness constraints. In contrast, Occam’s inversion utilizes a regularization parameter that explicitly balances data fit and model smoothness. Following Farquharson and Oldenburg^63^ an adaptive scheme is implemented α_n_ = max(cα_*n*−1_, α∗)^60^ where α_n_ is the regularization parameter at iteration n, α_*n*−1_ is the regularization parameter at the previous iteration, α∗ is optimal α value that minimizes the discrepancy function^58^ which simply means choosing the value of λ that minimizes the misfit, and c is restriction factor (0.01 ≤ c ≤ 0.5) controlling the decrease rate. The discrepancy principle involves selecting the value of λ that minimizes the misfit. A univariate search algorithm is used to find the minimum of χ(λ), starting from the λ value used in the previous iteration. This constrained reduction of α prevents both oversmoothing in early iterations and the development of artificial structures, while ensuring stable convergence toward geologically plausible models. Together, these approaches provide complementary advantages: Marquardt’s efficiency for rapid solution estimation and Occam’s robustness for producing physically meaningful, smooth models.

#### Joint inversion of TEM and RMT data

Joint inversion is a strategy designed to address two critical challenges in geophysical data interpretation: model equivalence and depth resolution limitations. Model equivalence arises from the inherent ambiguity of geophysical data, where multiple subsurface models can equally well explain the observed data. This ambiguity makes it difficult to determine the true subsurface structure. Depth resolution limitations occur when the depth of investigation of a single geophysical method is insufficient to accurately image deeper structures. Joint inversion overcomes these challenges by simultaneously inverting data from two or more complementary geophysical methods. By combining datasets with different sensitivities to subsurface properties and overlapping depth ranges, joint inversion improves model resolution, reduces ambiguity, and provides more reliable subsurface interpretations.

In the framework of joint inversion, the inversion algorithm basically works using the same schemes described for individual LM and Occam inversion algorithms. Different datasets, forward operators and Jacobian matrices are appended to the vectors^5,64^,

23 $$\:{\varvec{d}}_{joint}=\:\left[\begin{array}{c}{\varvec{d}}_{TEM}\\\:{\varvec{d}}_{RMT}\end{array}\right]\\{\varvec{F}}_{joint}=\:\left[\begin{array}{c}{\varvec{F}}_{TEM}\\\:{\varvec{F}}_{RMT}\end{array}\right]\\{\varvec{J}}_{joint}=\:\left[\begin{array}{c}{\varvec{J}}_{TEM}\\\:{\varvec{J}}_{RMT}\end{array}\right]$$

and the corresponding data misfit $\:{\varPhi\:}_{d}$ is defined as:

24 $$\Phi _{d} = \left\| {a_{1} \user2{W}_{{d,TEM}} \left( {\user2{d}_{{TEM}} - F_{{TEM}} \left( \user2{m} \right)} \right)} \right\|_{2}^{2} + \left\| {a_{2} \user2{W}_{{d,RMT}} \left( {\user2{d}_{{RMT}} - F_{{RMT}} \left( \user2{m} \right)} \right)} \right\|_{2}^{2}$$

where $\:{\varvec{W}}_{d,TEM}$​ and $\:{\varvec{W}}_{d,RMT}$​ are data weighting matrices for TEM and RMT. $\:{a}_{1}$ and $\:{a}_{2}$ are the data misfit balancing coefficients, which prevent the individual dataset to be dominant over other and ensure that both of them have a same influence on the overall misfit,

25 $$\:{a}_{i}=\:\sqrt{1/{N}_{i}}$$

where $\:{N}_{i}$ stands for the number of TEM and RMT data points. Therefore, a model that fits both datasets satisfies^64^,

26 $$\:\frac{{\chi\:}_{TEM}^{2}}{{N}_{TEM}}+\frac{{\chi\:}_{RMT}^{2}}{{N}_{RMT}}\approx\:1+1$$

**Fig. 2:**
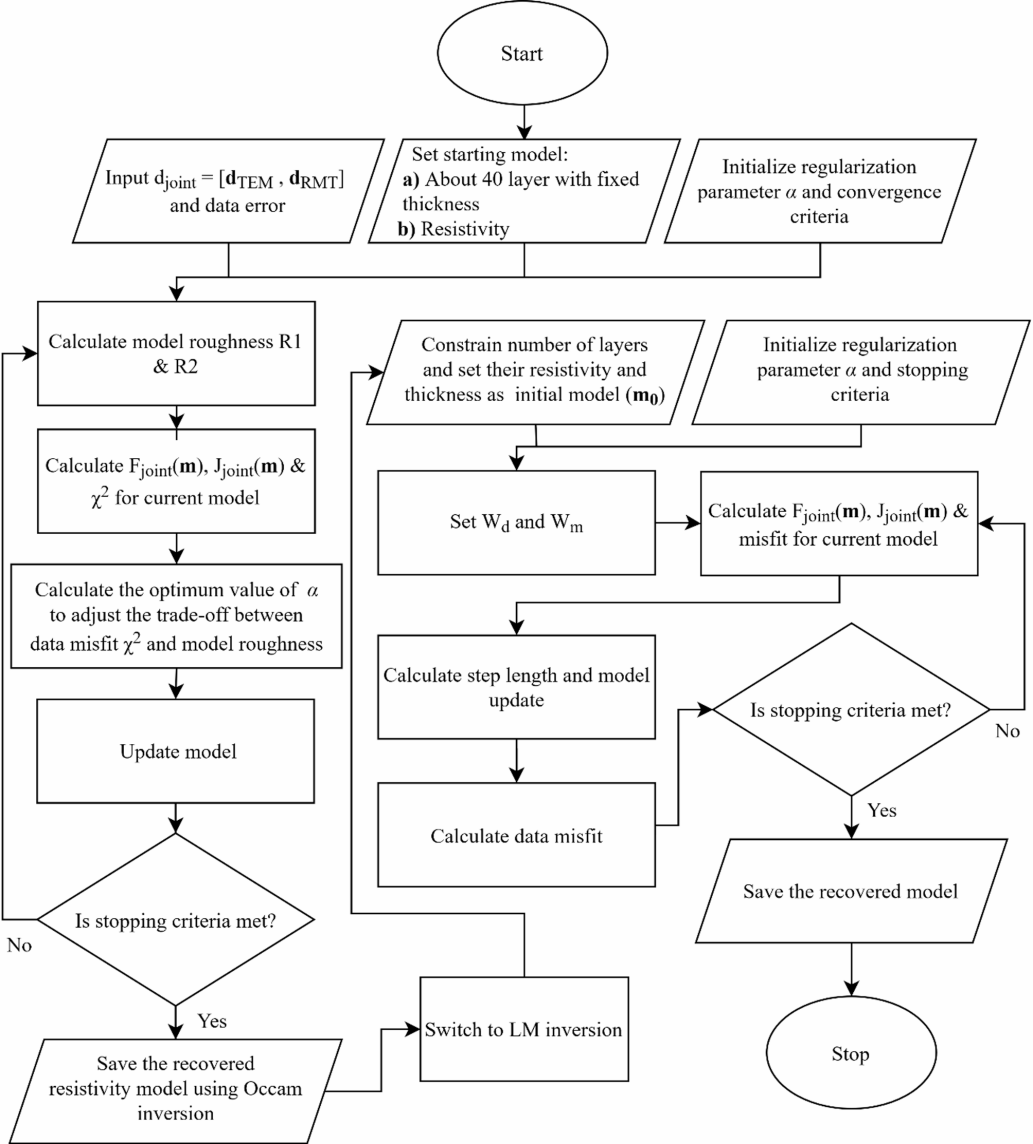
Flowchart of joint inversion of TEM and RMT data.

Figure 2 shows a flowchart illustrating the joint inversion process of TEM and RMT data. It begins with data input and model initialization, followed by iterative model updates that balance data misfit and model roughness using regularization. Two inversion approaches, Occam and LM, are employed to refine the resistivity model. The final recovered model is obtained once the stopping criteria are met, providing an improved subsurface characterization.

### Resolution and importance analysis

To evaluate the importance of the recovered model, the model resolution is examined. The resolution matrix ***R*** relates the estimated model ***m***_***est***_ obtained from inversion to the true model ***m***_***true***_^65,66^,

27 $$\:{\varvec{m}}_{est}=\varvec{R}{\varvec{m}}_{true}={\varvec{J}}^{*}\varvec{J}{\varvec{m}}_{true}=\varvec{V}{\varvec{V}}^{T}{\varvec{m}}_{true}$$

Where $\:{\varvec{J}}^{*}$ is the truncated generalized inverse derived from the singular value decomposition (SVD) of the Jacobian matrix $\:\varvec{J}=\varvec{U}\varvec{\varLambda\:}{\varvec{V}}^{\varvec{T}}$. U, Λ, and V are the orthogonal and diagonal matrices from the decomposition.

The importance of each model parameter can be quantified using the diagonal elements of the resolution matrix, expressed as *imp*_*j*_ = *R*_*jj*_ where 0 < *imp*_*j*_ < 1. A value of *R*_*jj*_ ​≈ 1 indicates that the parameter is well-resolved and independently constrained by the data, whereas *R*_*jj*_ ≪ 1 suggests that the parameter is poorly resolved, likely influenced by regularization or correlations with other parameters. This measure provides insight into the reliability and significance of each recovered parameter.

### Equivalent models

In practice, multiple models can fit geophysical data equally well due to the non-uniqueness and the complexity of the cost function, especially when data errors are considered. Poorly resolved parameters often lead to model equivalence, which is explored using a Monte Carlo approach where model parameters are randomly perturbed and accepted if they still fit the data^60^. This technique provides insights into model variability and non-linear parameter uncertainty, thus going beyond traditional linearized error analysis.

### Depth of investigation

The depth of investigation (DOI) is a key parameter in geophysical surveys, representing the maximum depth at which reliable information about the subsurface can be obtained. For TEM methods, the DOI is influenced by factors such as the transmitted current ($\:I$), the area of the transmitter loop ($\:A$), the average weighted effective resistivity ($\:\stackrel{-}{\rho\:}$​), and the noise level of the measured voltage ($\:{\eta\:}_{v}$​) (typically $\:{\eta\:}_{v}=\:$0.5 nV/m^2^). According to Spies^67^ the DOI of TEM can be estimated using:

28 $$\:DOI=0.55\:{\left(\frac{IA\stackrel{-}{\rho\:}}{{\eta\:}_{v}}\right)}^{1/5}$$

Alternatively, Meju^68^ proposed an expression based on the late-time response:

29 $$\:DOI=\frac{1}{2.3}\sqrt{\frac{2t\stackrel{-}{\rho\:}}{{\mu\:}_{0}}}$$

where $\:t$ is the last transient time and $\:{\mu\:}_{0}=$ 4$\:\pi\:\times\:{10}^{-7}$H/m is the magnetic permeability of free space. For the RMT method, the DOI is approximated as 1.5 times the skin depth ($\:SD$​), which is calculated using^67^:

30 $$\:SD=\sqrt{\frac{2}{\sigma\:{\mu\:}_{0}\omega\:}}=503\sqrt{\frac{{\rho\:}_{a}}{f}}$$

31 $$\:DOI\approx\:1.5\:SD\approx\:750\sqrt{\frac{{\rho\:}_{a}}{f}}$$

where $\:{\rho\:}_{a}$ is the apparent resistivity and $\:f$ is the frequency.

While these formulas provide useful estimates, care must be taken as DOI values may be overestimated. Alternatively, the divergence between Occam R1 and R2 can be used to qualitatively determine the effective DOI^69^. This divergence reflects zones of reduced model stability and sensitivity, offering a practical means to approximate the effective DOI for both methods.

### Synthetic modeling study

To validate the joint inversion approach, a synthetic modeling study was conducted using a simulated landfill model designed to represent the typical resistivity structure of landfill sites^70^. The model consisted of five distinct layers, each characterized by specific resistivity values and thicknesses, as detailed in Table 1.

**Table 1:** Synthetic landfill model parameters.

Layer no.	Resistivity (Ωm)	Thickness (m)
1	550	1.5
2	20	6.5
3	200	13
4	20	20
5	2.5	-

The uppermost layer, with a resistivity of 550 Ωm and a thickness of 1.5 m, represents a resistive soil layer commonly used in landfill engineering for isolation and protection. Beneath this, a conductive layer (20 Ωm) with a thickness of 6.5 m simulates the waste body, which typically contains leachate and decomposing materials that reduce resistivity. This waste body is underlain by a more resistive geological formation (200 Ωm) extending to a depth of 13 m, representing a compacted natural subsurface layer. Below this, another conductive layer (20 Ωm) with a thickness of 20 m suggests the presence of potential leachate infiltration or clay-rich sediments. The deepest layer, with a resistivity of 2.5 Ωm, represents a highly conductive saturated zone or clayey substratum, serving as the lower boundary of the model. TEM and RMT data were generated for this synthetic model, and the joint inversion approach was performed. To simulate a realistic field scenario, relative Gaussian noise with a standard deviation of 5% was added to both the TEM data and the apparent resistivity values of the RMT data, while a 2.5% noise was applied to the RMT phase data.

## Results and discussion

**Fig. 3:**
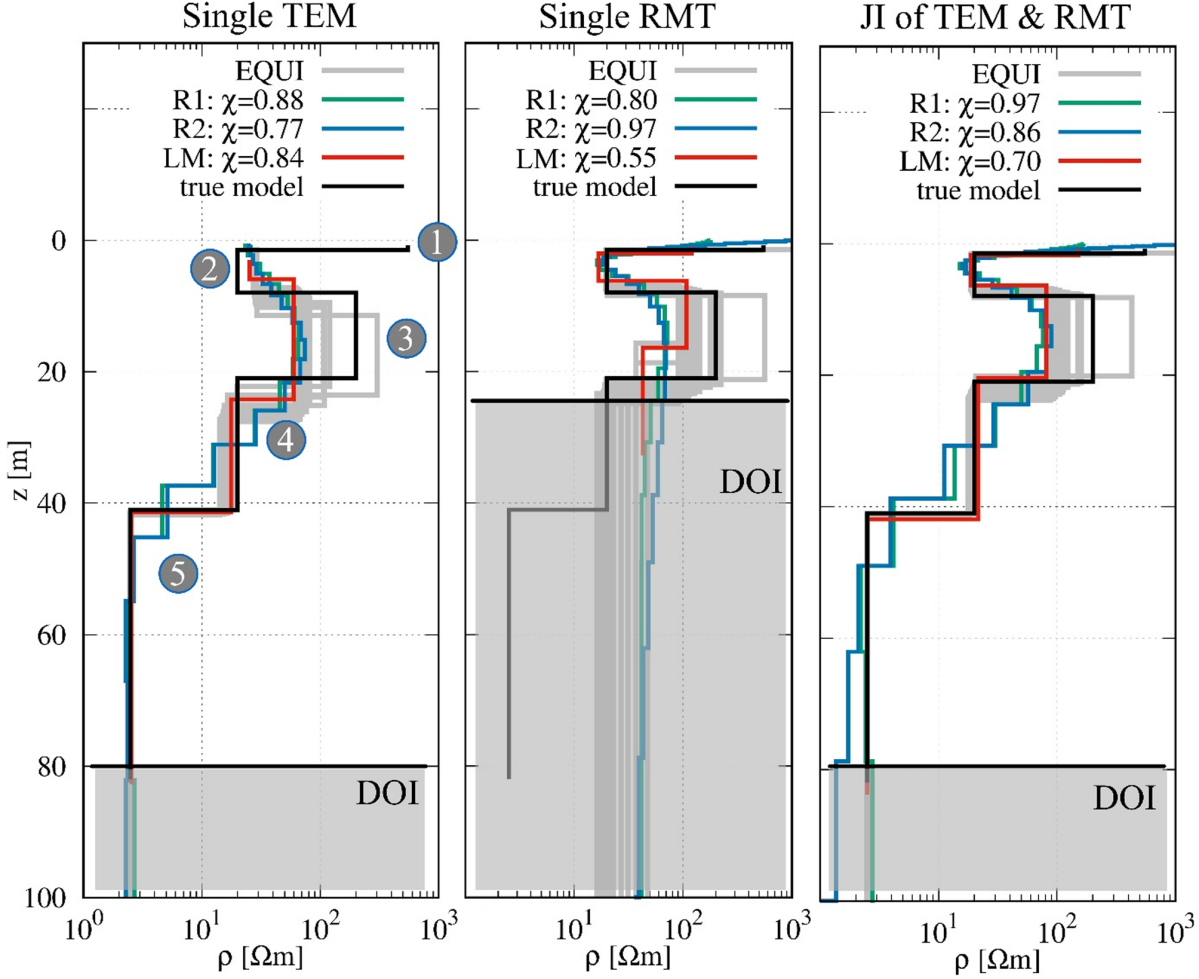
Results of the synthetic modeling study, showing the true model, the individual TEM and RMT inversions, and the joint inversion results. Numbers (1 to 5) in the figure refer to the true layers represented by black line (see also Table 1). The calculated DOI of TEM data is approximately 138 m (from Spies^67^) and 198 m (from Meju^68^), while the DOI of RMT data is around 44 m (from Spies^67^). We believe that they are over-estimated, therefore, the DOI is estimated qualitatively by evaluating the divergence between the Occam’s R₁ and R₂ inversion results.

The results of the 1D TEM, RMT, and joint inversions using synthetic data are presented in Fig. 3; Table 2 illustrating the strengths and limitations of each method in resolving the subsurface layers when compared to the true model. The RMT method effectively detects the first and second layers, particularly the waste body (layer 2), with resistivity value of 16.8 Ωm (true: 20 Ωm) and an importance of 0.96. However, it slightly underestimates the thickness of the second layer (4.18 m vs. true 6.5 m) and struggles with deeper structures. The third layer is poorly resolved, as seen in its low importance values (0.44 for resistivity and 0.64 for thickness), and the fourth layer is overestimated in resistivity (42.6 Ωm vs. true 20 Ωm) while its thickness is not resolved. Additionally, RMT does not identify the highly conductive fifth layer (2.5 Ωm) due to its limited depth of penetration.

In contrast, the TEM method is not able to detect the first layer but performs well in defining the deeper structures. While it captures the waste body (ρ_2_ = 24.6 Ωm vs. true 20 Ωm) with high confidence (importance = 0.95), the third layer is not well resolved, with its resistivity underestimated (ρ_3_ = 24.6 Ωm vs. true 200 Ωm) and thickness (5.77 m vs. true 13 m). However, deeper layers are more accurately identified, particularly the fourth and fifth layers, where the resistivity values (ρ_4_ = 17 Ωm vs. true 20 Ωm and ρ_5_ = 2.5 Ωm) and thickness estimations (h4 = 16.5 m vs. true 20 m) are reasonably well determined.

**Table 2:** Models (resistivity in Ωm and thickness in m) and importance (Imp) parameters of RMT, TEM, and joint inversions (JI) of the synthetic modeling study.

	TEM	Imp	RMT	Imp	JI	Imp
ρ_1_	-	-	119.80	0.63	150.91	0.35
ρ_2_	24.60	0.95	16.80	0.96	18.72	0.98
ρ_3_	56.90	0.86	107.70	0.44	80.92	0.88
ρ_4_	17.00	0.62	42.60	0.75	21.67	0.91
ρ_5_	2.50	0.99	-	-	2.51	1.00
h_1_	-	-	2.03	0.95	1.72	0.96
h_2_	5.77	0.54	4.18	0.85	4.61	0.87
h_3_	18.14	0.84	10.17	0.64	14.07	0.86
h_4_	16.50	0.89	-	-	21.51	0.96

**Fig. 4:**
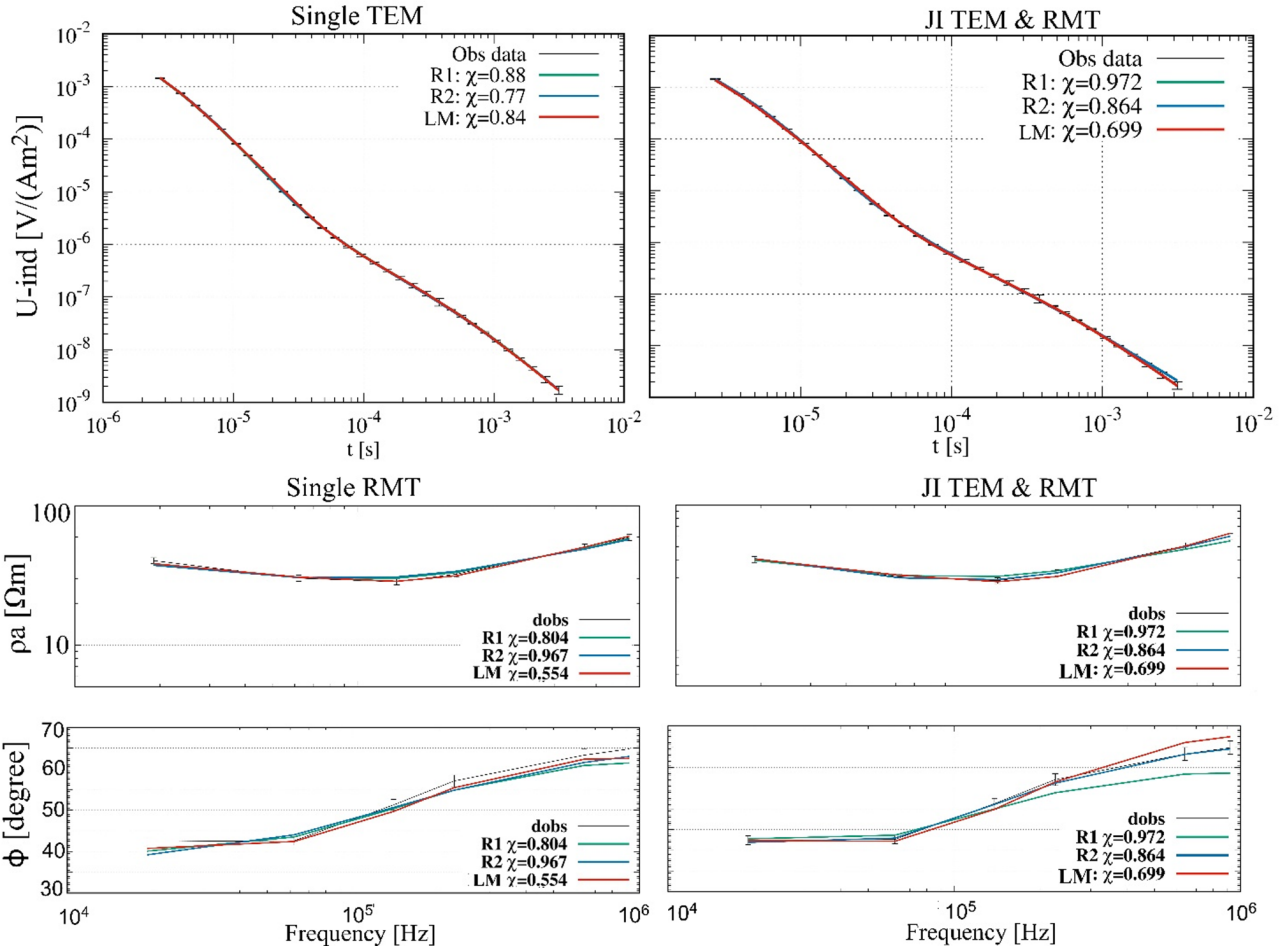
Data fitting results for single and jointly inverted TEM and RMT data from the synthetic modeling study. The plots show observed data alongside inversion results using Occam’s R1 and R2 approaches, as well as the LM method. The top row presents TEM data, while the bottom row displays RMT data, showing an overfitting of single and joint inversion approaches (χ < 1).

The joint inversion approach significantly improves the accuracy of the subsurface model by integrating the advantages of both methods. The first layer, which was only resolved by RMT, is now detected with a resistivity of 150.92 Ωm (true: 550 Ωm) and an improved thickness estimation (h1 = 1.72 m vs. true 1.5 m). The waste body (layer 2) is accurately resolved (ρ_2_ = 18.72 Ωm vs. true 20 Ωm, h_2_ = 4.61 m vs. true 6.5 m) with importance values of 0.98 and 0.87 for resistivity and thickness, respectively. The third layer’s resistivity is closer to the real model (ρ_3_ = 80.92 Ωm vs. true 200 Ωm, h_3_ = 14.07 m vs. true 13 m) compared to RMT alone, and the fourth layer is also well resolved with high importance values (0.91 for resistivity and 0.96 for thickness). The joint inversion accurately detects the deepest layer (ρ_5_ = 2.51 Ωm, importance = 1.0), which RMT alone failed to resolve. However, the fitting results presented in Fig. 4 further support these findings, demonstrating that the joint inversion approach provides a good agreement between observed and modeled data for both TEM and RMT. The χ values for all inversion approaches remain below 1, indicating an slight overfit. To assess the impact of data noise, we performed joint inversions using both the noise-contaminated and noise-free synthetic datasets. The resulting models were consistent, indicating robust inversion performance, with only slightly better χ values in the noise-free case as expected.

Overall, the joint inversion provides the most reliable resistivity model, successfully resolving the boundaries of the waste body and the underlying geological formations. The TEM method is advantageous for detecting deeper layers, while RMT is better suited for resolving the shallow layers, particularly the waste body. By combining the strengths of both methods, the joint inversion provides a more accurate and complete picture of the subsurface. This demonstrates the importance of integrating different geophysical techniques to overcome their individual limitations and achieve more precise subsurface characterization.

While Occam inversion does not require a detailed initial model, it typically begins from a uniform half-space, which, although not critical due to the smoothing constraints, is still necessary to initiate the inversion. In contrast, the quality of the LM inversion result depends heavily on the choice of the initial model, which is typically derived from the Occam inversion output. Poorly chosen initial models can lead to suboptimal or inaccurate results, as the inversion process may converge to a local minimum that does not accurately represent the true subsurface structure. This limitation is particularly critical in heterogeneous environments like landfills, where subsurface properties can vary significantly.

## Application to landfill characterization

Landfills present significant challenges for subsurface characterization due to their heterogeneous composition and contamination risks. Many old sites lack containment systems, leading to leachate infiltration and groundwater pollution^71–75^. Traditional methods like drilling and sampling are costly, time-consuming, provide limited insights, and may create additional pathways for leakage or fluid migration^76,77^. Geophysical techniques, particularly TEM and RMT, offer non-invasive, cost-effective alternatives for imaging landfill boundaries, buried waste, and contamination plumes^23,39–41^.

**Fig. 5:**
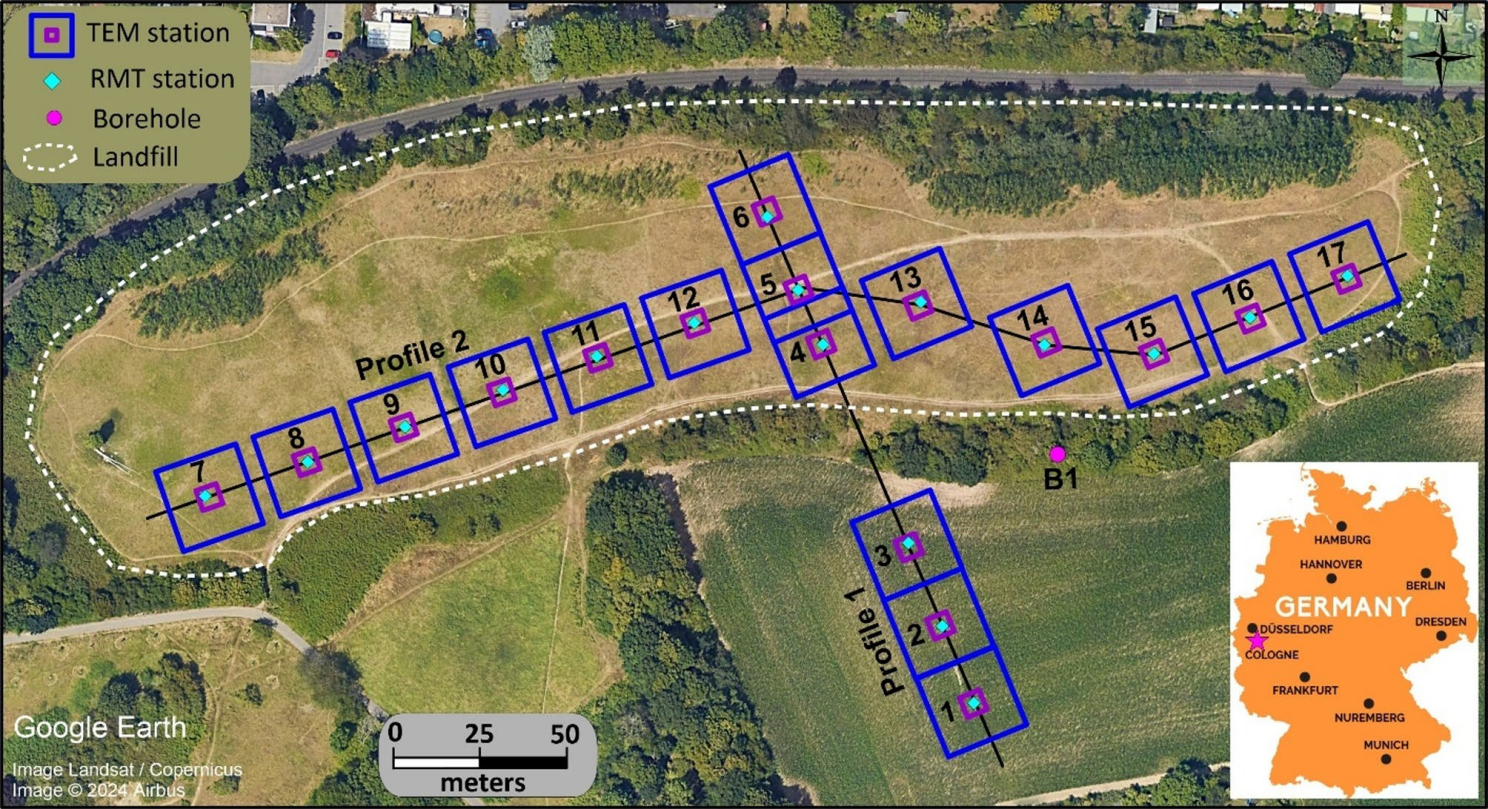
A map displaying the distribution of TEM and RMT stations and the location of borehole B1 at the Weidenpesch landfill in Cologne, Germany. The dumpsite is outlined with a dashed white line. Imagery ©2024 Maxar Technologies, utilized in compliance with the terms of service of Google Maps/Earth for academic research purposes.

The joint inversion of TEM and RMT data was applied to the Weidenpesch landfill site (Fig. 5) in Cologne, Germany. The landfill initially established as a sand and gravel pit in the late 1950s, transitioned into a landfill for domestic and industrial waste in 1966. Over the following decade, materials such as construction debris, household waste, soil, and stone dust were disposed of there. When the site closed in 1976, it was covered by a thin layer of silty fine sand, with a thickness of 0.5–2.5 m^78^. The subsurface lithology consists of Pleistocene and Holocene floodplain sediments resting on a Pleistocene gravelly sand layer, with deeper Tertiary deposits composed of clay, brown coal, and sand^70,79,80^. Borehole data from surrounding areas show the Holocene silt and clay layers reach up to 2.8 m in thickness, while the Pleistocene gravel and sand layers range from 17 to 25 m. Beneath this lies the Oligocene base, made up of fine sand, sandy coal, silty coal, and brown coal^81,82^.

**Fig. 6:**
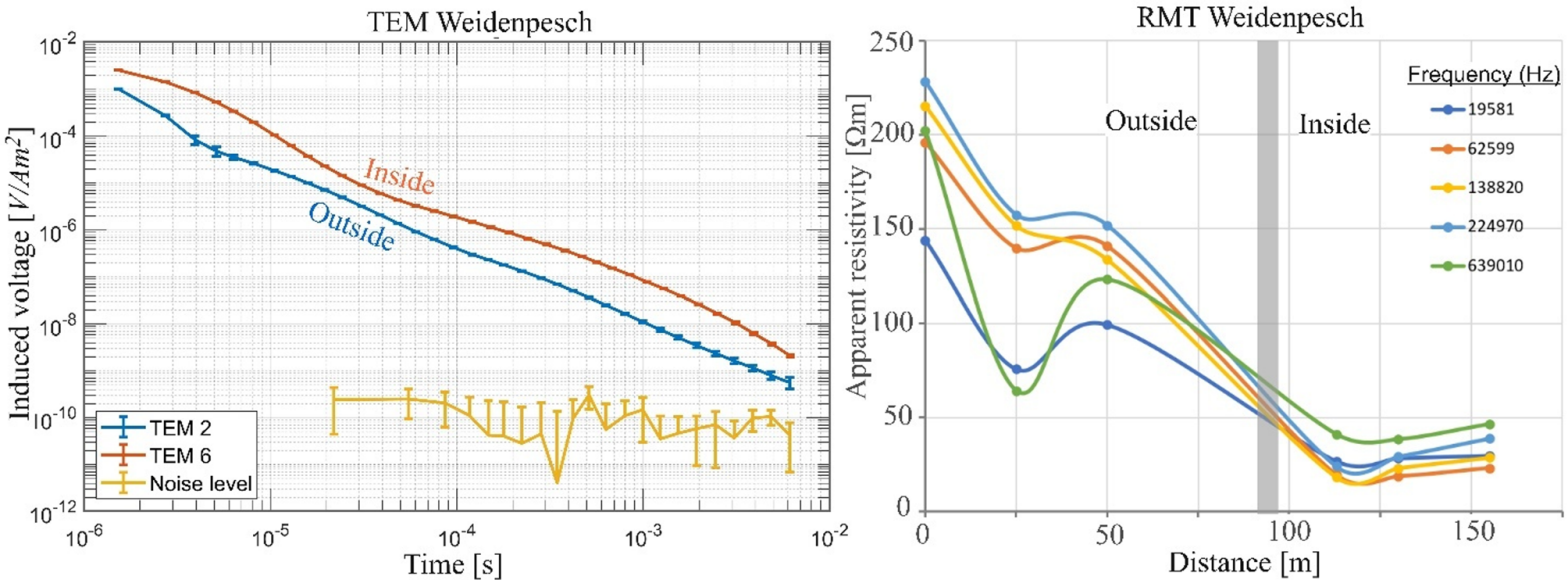
Left: Examples of TEM transients measured outside (TEM 2) and inside (TEM 6) the landfill, illustrating the contrast in response. The noise level in the area is also presented. Right: Apparent resistivity curves obtained from the RMT survey along profile 1, which intersects the landfill at approximately 95 m, shown for multiple frequencies.

The TEM and RMT data were measured along two profiles at 17 stations as shown in Fig. 5. The TEM survey was conducted using a central loop setup. The transmitter loop has an area of 25 × 25 m², while the receiver loop is 5 × 5 m² inside the landfill and 10 × 10 m² outside it. Data acquisition was carried out utilizing the Zonge GDP-3224 system. The NanoTEM measurements, covering a time range of 1.5 × 10^−3^ to 1 ms, were carried out using a 3 A current, whereas the ZeroTEM measurements, covering 0.1 to 6 ms, utilized a 10 A current. For each station, NanoTEM and ZeroTEM transients were combined into a single dataset, with the NanoTEM ramp time (2.7 µs) taken into account during the inversion process. Noise levels were also measured, showing that the recorded signals were clearly above the noise threshold, particularly in the 1 to 10 ms time window. The RMT-F equipment from the University of Cologne^43^ was used to conduct a RMT survey at approximately the same locations of the TEM stations (Fig. 5). Figure 6 provides examples of TEM and RMT data acquired inside and outside the landfill. The results show a clear change in behaviour within the landfill, where the subsurface becomes noticeably more conductive as expected in the presence of waste material or leachate.

In contrast to the eastern section, the resistivity in the western and central areas is lower, likely due to the heterogeneous composition of the buried materials. The waste body in the eastern part of the landfill exhibits an average resistivity value of 50 Ωm, while the resistivity in the central and western parts tends to be lower (an average value of 10 Ωm), reflecting increased moisture content and variability in material composition^70^. Accordingly, we present two examples of joint inversion results of TEM and RMT data: one from the eastern part of the landfill (station 17) and another one from the central part (station 11).

**Fig. 7:**
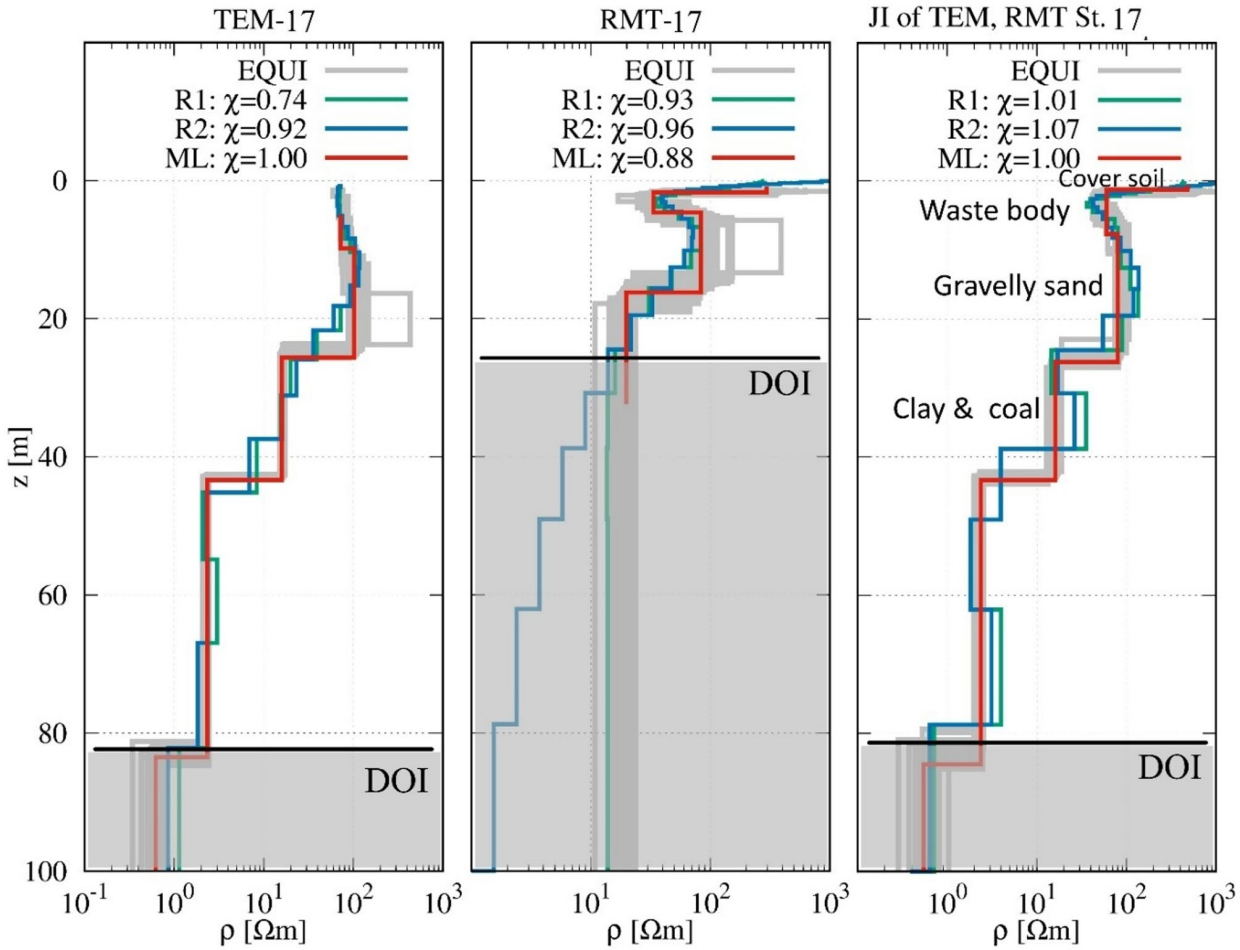
Comparison between resistivity models based on the results of 1D RMT, TEM, and joint inversions at station 17.

Figure 7 presents the 1D inversion and interpretation for station 17 (Fig. 5), showing results from Occam inversions with different roughness constraints (R1 and R2), LM inversions, and equivalent (EQ) models for single and joint inversion data. The first layer is a thin soil cover with a resistivity of 550.8 Ωm and a thickness of 1.43 m. This layer represents the surface material, likely consisting of compacted soil or mixed landfill cover. The second layer corresponds to the waste body, which has a resistivity of 56.9 Ωm and extends to a depth of approximately 8.63 m. This layer is characterized by a heterogeneous mixture of municipal and industrial waste. Beneath the waste body, the third layer is a moderately resistive formation with a resistivity of 154.7 Ωm and a thickness of 12.42 m, reaching a depth of approximately 21.06 m. This layer likely represents a sandy or gravelly deposit. The fourth layer is a more conductive unit with a resistivity of 19.8 Ωm and a thickness of 20.02 m, indicating a transition zone, possibly composed of silty or clay-rich sediments with moderate water saturation. The fifth and deepest layer is a highly conductive formation with a resistivity of 2.79 Ωm, suggesting the presence of wet lignite (brown coal) or clay, consistent with geological data^70^. The joint inversion of TEM and RMT data provided a well-constrained subsurface model, achieving good agreement with the known stratigraphy. Compared to individual inversions, the joint approach enhances the resolution of deeper layers while maintaining accuracy in identifying the waste body. The high importance values, particularly 0.98 for the waste body and 1.0 for the deepest conductive layer, confirm the reliability of the inversion results. Table 3 presents the resistivity models, layer thicknesses, and associated importance parameters derived from the RMT, TEM, and joint inversion (JI) results for station 17.

**Table 3:** Models (resistivity in Ωm and thickness in m) and importance parameters of RMT, TEM, and joint inversions (JI) for station 17.

	TEM	Imp	RMT	Imp	JI	Imp
ρ_1_	-	-	296.00	0.88	479	0.20
ρ_2_	72.00	0.99	33.40	0.99	60.00	0.99
ρ_3_	102.00	0.91	83.43	0.98	80.00	0.99
ρ_4_	15.90	0.97	19.73	0.99	16.00	1.00
ρ_5_	2.33	0.98	-	-	2.37	1.00
ρ_6_	0.62	0.40	-	-	0.6	0.98
h_1_	-	-	1.73	0.97	1.30	0.95
h_2_	9.82	0.64	2.87	1.00	6.43	0.70
h_3_	15.82	0.84	11.60	0.96	18.55	0.96
h_4_	17.73	0.99	-	-	17.11	1.00
h_5_	40.15	1.00	-	-	41.17	1.00

**Fig. 8:**
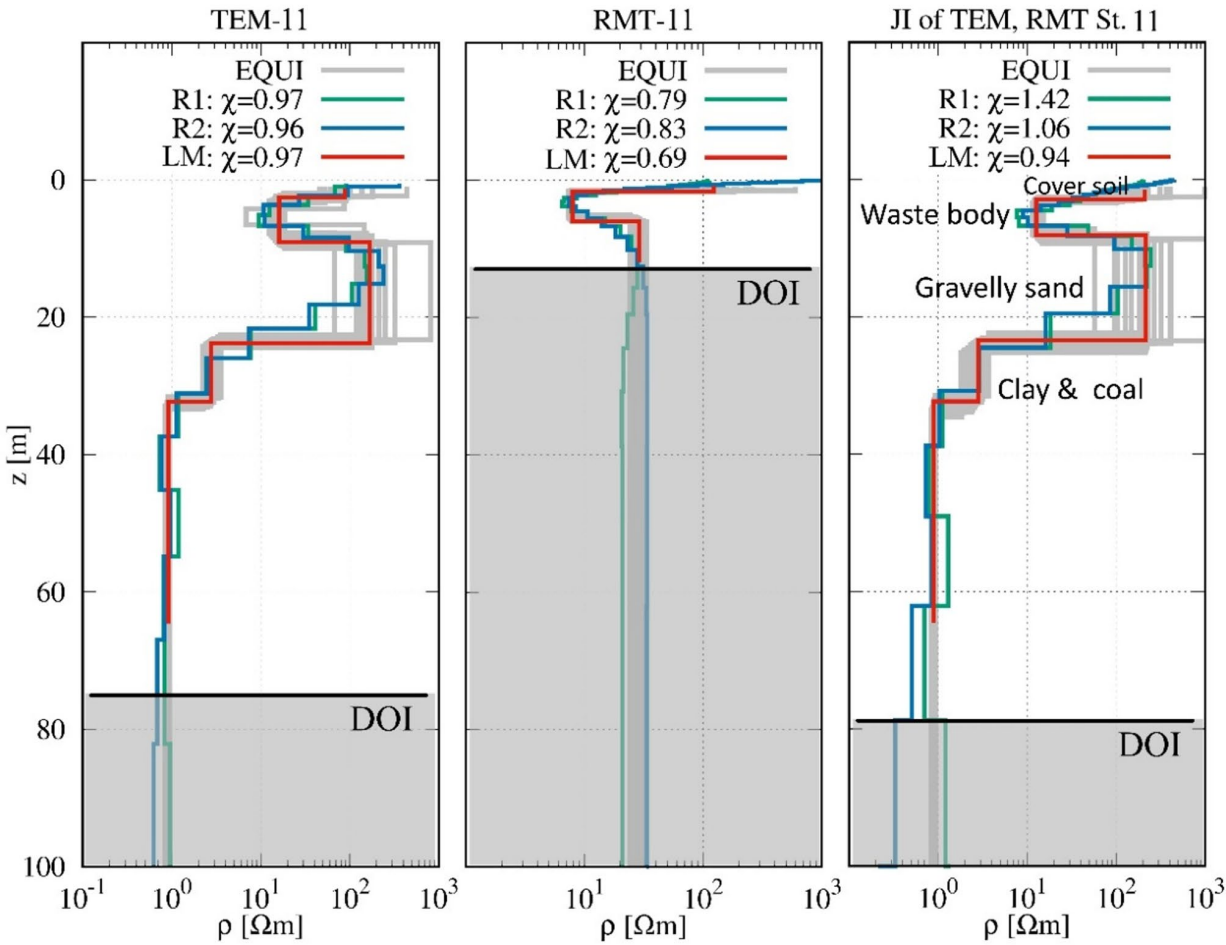
Comparison of the resistivity models obtained from the results of RMT, TEM, and joint inversions at station 11.

Figure 8 illustrates the 1D joint inversion results for station 11, located in the central part of the landfill. The first layer, with a resistivity of 209.9 Ωm and a thickness of 2.86 m, represents a soil cover. Beneath it, the waste body is identified with a lower resistivity of 12.7 Ωm and a thickness of 5.22 m, reflecting higher moisture content or increased organic material compared to the eastern section. The third layer, a more resistive zone (214.6 Ωm), extends 15.3 m and likely consists of sand or gravel. Deeper, the fourth layer is a conductive deposit (2.85 Ωm) with a thickness of 8.91 m, indicative of coal or clay-rich sediments. At the base, a layer shows extremely low resistivity (0.9 Ωm), pointing to the presence of saturated clay or lignite. The joint inversion effectively maps these layers with high confidence, as indicated by the importance values, particularly 0.96 for the waste body and 0.99 for the lowest layer, reinforcing the robustness of the model.

**Fig. 9:**
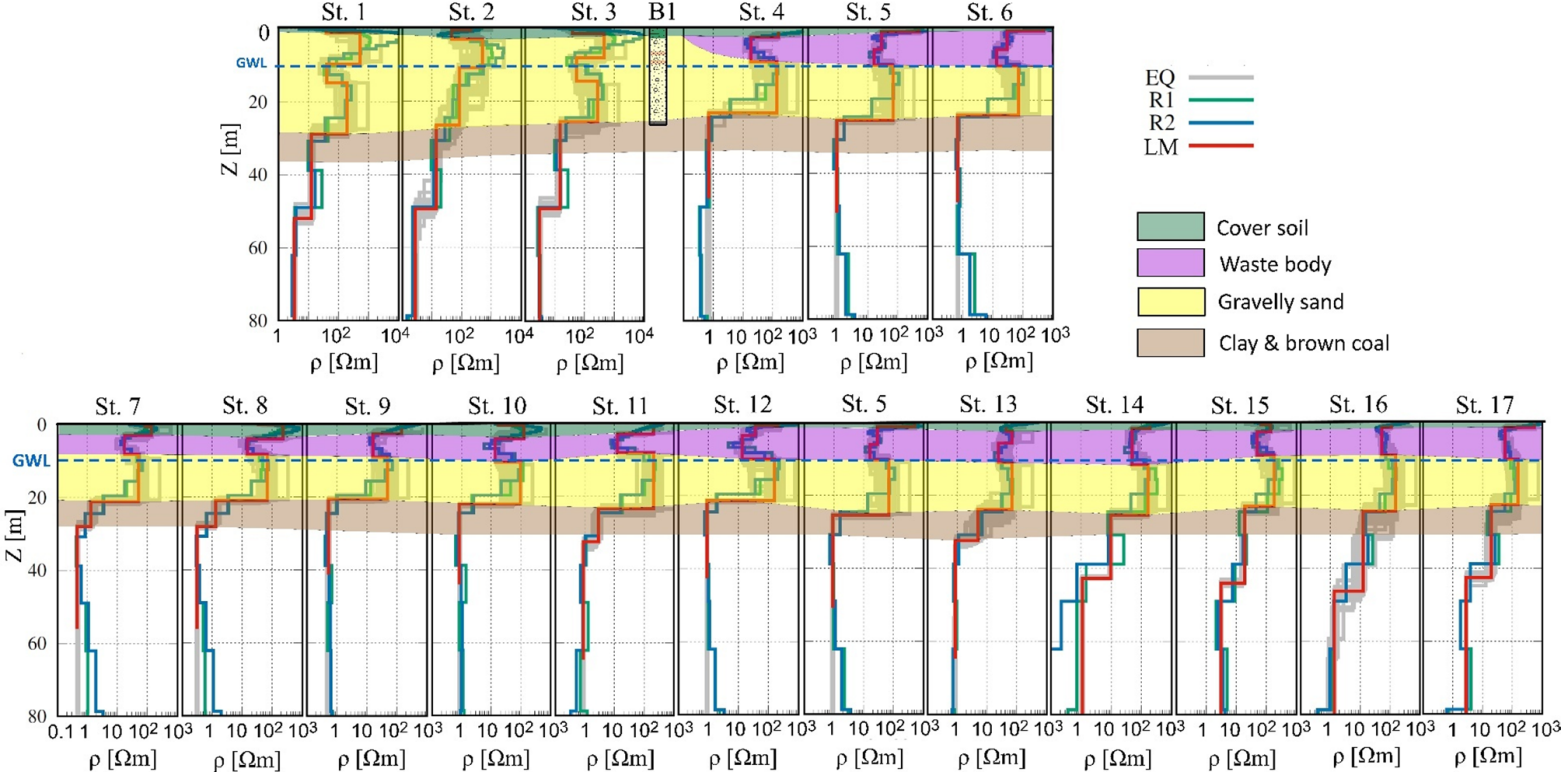
Results of 1D joint inversion of TEM and RMT along profile 1 (upper panel) and profile 2 (lower panel). The lithology of borehole W2 (Fig 0.1) is presented between stations 3 and 4. Dashed blue line refer to the groundwater level (GWL) in the area.

The joint inversion results for all 17 stations are presented along Profile 1 (stations 1–6) and Profile 2 (stations 7–12, 13, and 14–17) (Fig. 9). The inversion reveals the presence of three primary geological layers in addition to the waste body. A resistive soil cover, with a thickness of up to 2.5 m, overlays the subsurface. Beneath this, a gravelly sand layer extends to varying depths, reaching approximately 20 m below stations 7, 8, and 9, and up to 27 m beneath station 1. This is followed by a highly conductive layer, interpreted as clay and brown coal. Within the gravelly sand, the waste body is observed as a conductive zone extending to depths of up to 10 m. Its resistivity varies, averaging 12 Ωm in the western and central sections of the landfill, increasing to 55 Ωm in the eastern part. Borehole B1, located between stations 3 and 4, provides direct validation of the joint inversion results, showing good agreement with the interpreted subsurface layers. Additionally, the decrease in resistivity observed in the gravelly sand beneath the landfill, from several hundred ohm-meters outside the landfill to around 100 Ωm directly below it, may indicate a possible contaminant leaching. This interpretation is supported by the hydrogeological setting, where the waste body is in direct contact with the unconfined aquifer, and groundwater occurs at an average depth of approximately 10 m, as shown in both geophysical profiles (Fig. 9) and supported by historical observations reported by Kotowski and Fröhlich^81^. Such conditions enhance the likelihood of leachate migration, particularly in zones of reduced resistivity. Moreover, the findings align well with recent Electrical Resistivity Tomography (ERT) investigations conducted by Ibraheem et al.^70^.

Given that our measurements at the Weidenpesch landfill were conducted away from the landfill edges, where lateral resistivity contrasts are minimal, and that borehole data and geological information indicate a laterally continuous, layered subsurface^70,83,84^, the use of a 1D inversion approach is well justified for this site. For TEM data, particularly in the central loop configuration, the induced currents are focused directly beneath the transmitter, effectively minimizing the influence of 2D or 3D structures. This makes 1D inversion generally suitable, except near strong lateral resistivity contrasts, such as at the boundaries of waste bodies, or in areas with significant topographic variation. Moreover, measuring the vertical component of the magnetic field at the loop centre further reduces sensitivity to lateral inhomogeneities^51^. Although RMT is inherently more sensitive to lateral variations due to its broader plane-wave response, the consistent layering and off-boundary measurement locations at the Weidenpesch site support the applicability of 1D inversion for RMT data as well.

## Conclusions

The joint inversion of TEM and RMT data has proven to be a highly effective approach for improving the accuracy and resolution of subsurface models, particularly in complex and heterogeneous environments such as landfills. By integrating the complementary strengths of TEM and RMT, where TEM excels in imaging deeper structures and RMT provides high-resolution data for shallow layers, joint inversion significantly reduces model ambiguity and enhances the reliability of subsurface interpretations. The synthetic modeling study validated the methodology, showing that joint inversion outperforms individual inversions in resolving both shallow and deep subsurface features, including the identification of the waste body.

The application of this approach to the Weidenpesch landfill in Cologne, Germany, further demonstrated its practical utility, revealing detailed subsurface structures with high confidence. In the individual inversion results, the RMT method effectively imaged the shallow subsurface, including the cover soil and the waste body. However, it lacked sufficient resolution to clearly define the bottom of the waste body and the underlying gravelly sand layer. In contrast, the TEM method was less sensitive to the shallow structures, as it failed to resolve the cover soil and the upper part of the waste body, but it provided reliable imaging of the deeper subsurface, particularly the base of the waste body and the underlying layers. By combining the complementary sensitivities of both methods, the joint inversion yielded a significantly improved model. It successfully resolved the entire section, from the near-surface cover layers down to the deeper geological units, providing a consistent and well-constrained interpretation of the landfill site.

The ability to detect variations in electrical resistivity across different sections of the landfill provided valuable insights into the distribution of waste materials and potential leachate leakage, which are critical for environmental monitoring and risk assessment. The findings of this research have significant implications for environmental geophysics, particularly in the context of landfill management and contamination monitoring. By providing a non-invasive and cost-effective method for subsurface characterization, joint inversion of TEM and RMT data can support more informed decision-making in environmental risk assessment and remediation efforts. One limitation of this study is the exclusion of induced polarization (IP) effects in the TEM data, which may be significant in waste-rich, conductive environments.

## Data Availability

Datasets generated during the current study are available from the corresponding author on reasonable request. The EMUPLUS inversion code used in this study is available upon reasonable request under the institutional license agreement with the University of Cologne.
